# Pneumonia due to *Achromobacter xylosoxidans* with a chronic course resembling non‐tuberculous mycobacterial infection

**DOI:** 10.1002/rcr2.1287

**Published:** 2024-02-01

**Authors:** Tomoko Kotani, Toshiaki Inazaki, Hajime Kasai, Shintaro Rakuman, Kenichi Suzuki, Takashi Urushibara

**Affiliations:** ^1^ Department of Respirology Kimitsu Chuo Hospital Kisarazu Japan; ^2^ Department of Respirology Graduate School of Medicine, Chiba University Chiba Japan; ^3^ Department of Medical Education Graduate School of Medicine, Chiba University Chiba Japan; ^4^ Health Professional Development Center Chiba University Hospital Chiba Japan

**Keywords:** *Achromobacter xylosoxidans*, antibiotics, chronic pneumonia, colonization, non‐tuberculosis mycobacterium

## Abstract

*Achromobacter xylosoxidans* is a common bacterium that rarely causes pneumonia. Determining whether *A. xylosoxidans* is the cause of lung infection in patients suspected of having chronic infectious lung disease is challenging because it can present with colonization. We report a case of a 56‐year‐old immunocompetent woman suspected of having non‐tuberculous mycobacteria (NTM) infection on imaging examination and monitored for 3 years. Sputum examinations revealed *A. xylosoxidans* several times, and it was determined to be a colonization. *A. xylosoxidans* was isolated from bronchial lavage fluid and aspirated sputum, but no evidence of NTM was observed. She was diagnosed with *A. xylosoxidans* infection and given ceftazidime for 2 weeks. Her symptoms and imaging findings improved rapidly after treatment, without recurrences. *A. xylosoxidans* rarely causes chronic lower respiratory tract infections similar to NTM in immunocompetent patients. *A. xylosoxidans* may be a target for treatment when detected in lower respiratory tract specimens.

## Graphical abstract

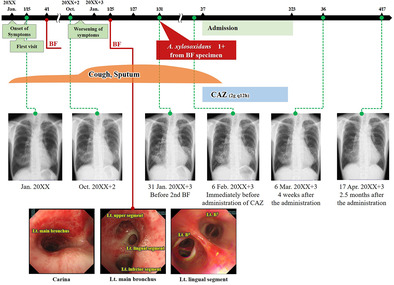

## INTRODUCTION

*Achromobacter xylosoxidans* is a gram‐negative bacterium usually found in bodies of water.^1^ It frequently causes acute infections, including those related to catheter use and pneumonia, particularly during hospitalization.^2^ Although multiple cases of infection have been reported in patients with cystic fibrosis (CF), cases of long‐term lung infections in individuals with other lung diseases are rarely documented.^3^ Various antibacterial drugs, such as ceftazidime and levofloxacin, are used in the treatment of *A. xylosoxidans* infections.^4^ However, a standardized treatment protocol for *A. xylosoxidans* infection has not yet been established.

Herein, we present a case of pneumonia caused by *A. xylosoxidans* that exhibited a chronic progression similar to that of non‐tuberculous mycobacteria (NTM) infections.

## CASE REPORT

**FIGURE 1:**
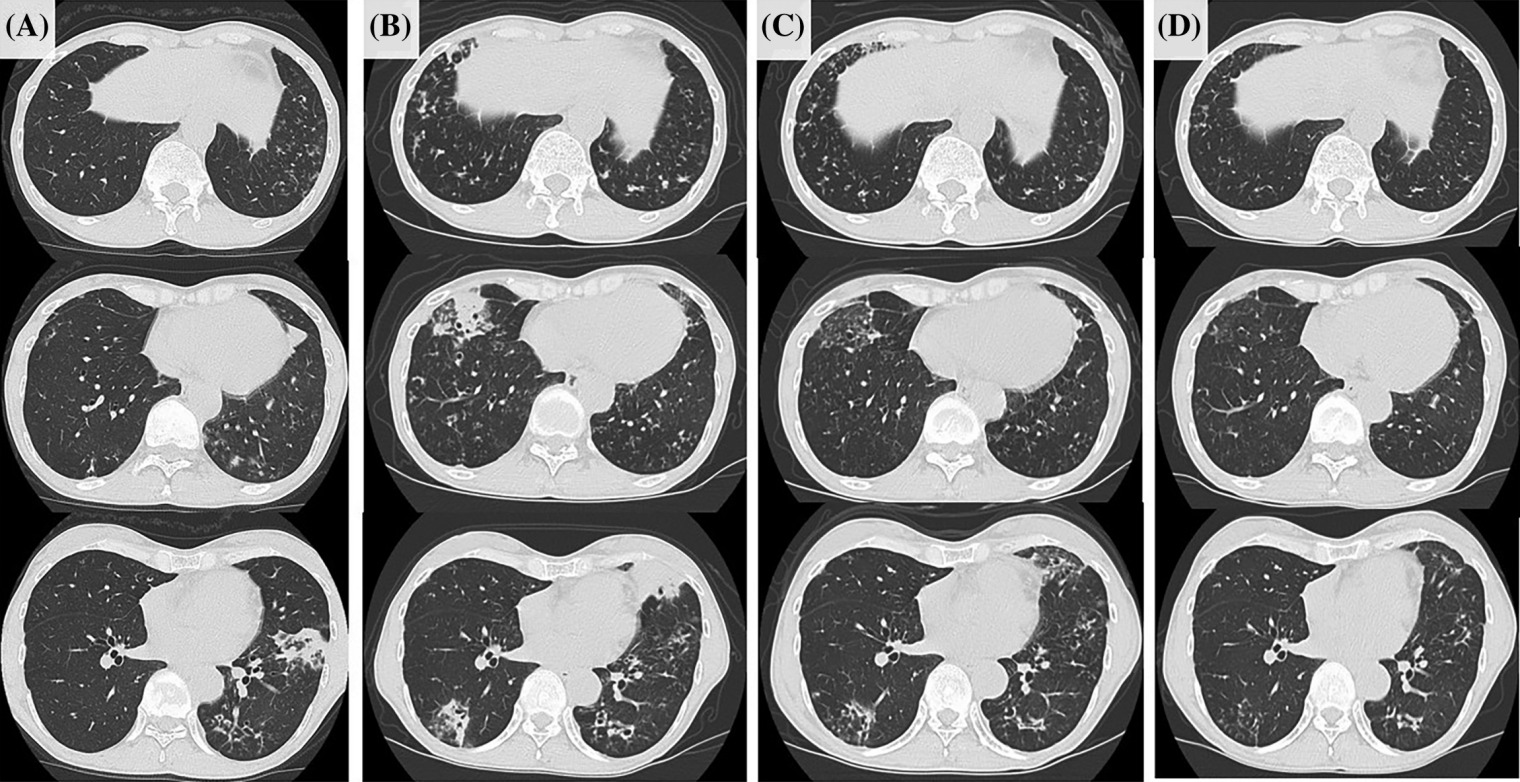
(A) Chest computed tomography (CT) revealed bronchiectasis and a small nodular shadow along the airways in both lungs at the first visit. (B) Chest CT before introduction of ceftazidime (CAZ) shows multiple nodules and consolidations in addition to a gradual exacerbation of the bronchiectasis and small nodular shadow along the airway in both lungs, which were detected 3 years ago. (C) Four weeks after the administration of CAZ, the nodular and infiltrative shadows decreased. (D) Two and a half months later, these findings had further improved.

A 56‐year‐old woman with haemoptysis visited our hospital in January 20XX. Chest computed tomography (CT) revealed bronchiectasis and a small nodular shadow and infiltrative shadows along the airways, mainly in the bilateral lower lobes (Figure 1A). She had a history of pleurisy when she was 42 years old. She has no prior remarkable medical history that suggests immunodeficiency. As an NTM infection was suspected, the patient underwent bronchoscopy. However, acid‐fast bacteria were not detected from sputum obtained from the bronchi. *A. xylosoxidans* was detected in the general bacterial culture of the same specimen (100 colonies). She was monitored through multiple sputum examinations for acid‐fast bacterium sputum, but no acid‐fast bacterium was detected. Sputum examination for general bacteria was performed once a year over a 3‐year period, which revealed *A. xylosoxidans* (Geckler 5, 10 colonies). *A. xylosoxidans* has been rarely reported to cause chronic infection, and the patient did not have any respiratory symptoms other than haemoptysis. No other findings suggest acute respiratory tract infection. Thus, we considered it a colonization.

**FIGURE 2:**
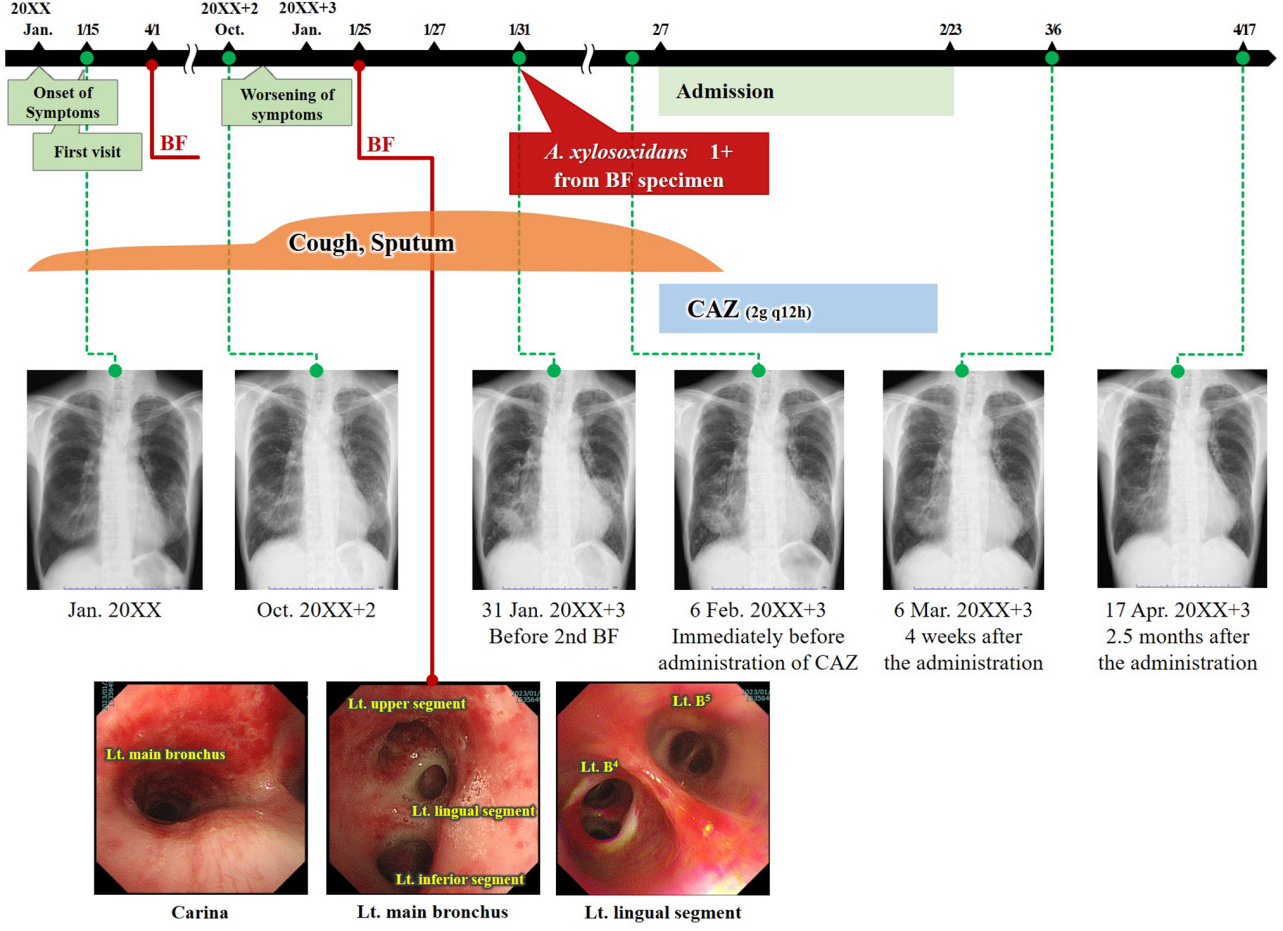
Clinical course of the patient. BF, bronchoscopy; CAZ, ceftazidime; Lt, left.

In January 20XX + 3, her symptoms, including cough and sputum, gradually worsened without any triggers such as upper respiratory tract infections. During a routine visit, chest radiography revealed worsening of the shadows in the lungs (Figure 2). Chest CT also showed a new shadow, in addition to the gradual exacerbation of the pre‐existing shadow (Figure 1B). Because of suspicion of NTM exacerbation, she was admitted for bronchoscopy to identify the species of NTM. On admission, the patient's weight and height were 37 kg and 156 cm, respectively. Her vital signs were normal, except for a low‐grade fever. Chest auscultation revealed coarse bilateral crackles in the thorax. The patient had no disease suggestive of immunosuppression. The patient was not exposed to any soil that could have triggered an NTM infection. She had also no contact, in the form of activities related to occupation, hobbies, or other reasons, with bodies of water that appeared to be contaminated by *A. xylosoxidans*. Furthermore, she was not a medical professional and had no history of prolonged hospitalization. Blood tests revealed a mild increase in C‐reactive protein (2.00 mg/dL). The immunoglobulin levels were within the normal range, and human immunodeficiency virus infection was negative. Bronchoscopy revealed excessive yellowish white sputum in the left bronchi (Figure 2). *A. xylosoxidans* was detected in the bronchial lavage fluid (2+) and in the aspirated sputum (1+). However, no acid‐fast bacteria were detected in these samples. She was diagnosed with chronic *A. xylosoxidans* infection. Antimicrobial therapy with ceftazidime (2 g/day) was administered via continuous infusion for 14 days, followed by oral erythromycin 200 mg/day as a low‐dose macrolide. After ceftazidime administration, her symptoms and imaging findings improved rapidly and did not worsen for 5 months after discontinuation of ceftazidime (Figures 1C,D and 2).

## DISCUSSION

The present case presents two notable clinical findings. First, *A. xylosoxidans* causes chronic pulmonary infections. Second, general bacterial culture might be important even for patients with imaging findings and progression similar to NTM because there could be patients with chronic infection mimicking *A. xylosoxidans* infection.

*A. xylosoxidans* has emerged as a potential causative agent of chronic pulmonary infections, even in individuals without CF. Notably, *A. xylosoxidans*‐induced pneumonia is uncommon in adults without systemic or airway immunodeficiencies.^3^ In our review of previously reported cases similar to ours, we identified six cases of pneumonia with *A. xylosoxidans* (Table 1).^5^, ^6^, ^7^, ^8^, ^9^, ^10^ These cases, along with our case, primarily involved relatively young patients (median age, 52.5 years; range, 45–76 years), with no sex disparity (men/women, 3:4). Bronchiectasis emerged as a prevalent comorbidity (four cases, 57%). *A. xylosoxidans* was most commonly detected in the bronchoalveolar lavage fluid (five cases, 71%), but some were detected in sputum (two cases, 29%). Although various antibiotics, such as ceftazidime, carbapenem, and quinolones, were used, the antimicrobial sensitivity for *A. xylosoxidans* was good in all cases. Typically, treatment durations extend to approximately 2 weeks, yielding relatively positive prognoses.

**TABLE 1:** Previously reported cases of pneumonia with *Achromobacter xylosoxidans* (5–10).

No	Report (year)	Age	Sex	Clinical syndrome	Comorbid conditions	Isolation specimen	Drug sensitivity	Antibiotic of choice	Drug use period (days)
1	Chandrasekaran et al. (2012)	NA	NA	Pneumonia	Colon cancer	Tracheal aspirate	Ceftazidime, piperacillin/tazobactam, cefoperazone‐sulbactam, levofloxacin, imipenem, meropenem, tigecycline	PIPC/TAZ	14
2	Atalay et al. (2012)	50	Female	Pneumonia	Adrenal insufficiency, caused by pneumonia	BAL	Ceftazidime, ciprofloxacin, ofloxacin, piperacillin/tazobactam, cefoperazone/sulbactam	CPFX	14
3	Karanth et al. (2014)	55	Male	Bronchial pneumonia	Bronchiectasis	BAL	NA	NA	NA
4	Bharadiya et al. (2014)	48	Male	Pneumonia	History of tuberculosis	Sputum	Meropenem, imipenem, piperacillin, ticarcillin, trimethoprim‐sulfamethoxazole, third‐generation cephalosporins	MEPM	14
5	Awadh et al. (2017)	45	Female	Pneumonia	Bronchiectasis, asthma, gastroesophageal reflux disease	BAL	Amikacin, cefepime, ceftazidime, gentamicin, levofloxacin, meropenem, piperacillin/tazobactam, tobramycin, trimethoprim/sulfamethoxazole	LVFX	42
6	Stepman et al. (2020)	76	Female	Pneumonia	MAI‐colonization, bronchiectasis	BAL	Tobramycin, amikacin, piperacillin/tazobactam, meropenem	MEPM	14
7	Present case	56	Female	Pneumonia	Bronchiectasis	Sputum BAL	Piperacilin, ceftazidime, imipenem/cilastatin, meropenem, minocycline, levofloxacin, trimethoprim/sulfamethoxazole	CAZ	14

Abbreviations: BAL, bronchial lavage; CAZ, ceftazidime; CPFX, ciprofloxacin; LVFX, levofloxacin; MAC, Mycobacterium avium complex; MAI, mycobacterium avium‐intracellulare; MEPM, meropenem; NA, not applicable; PIPC/TAZ, Piperacillin/Tazobactam.

Determining whether *A. xylosoxidans* detected in airway specimens is the cause of lung infection in patients suspected of having chronic infectious lung disease can be challenging because it can cause colonization. *A. xylosoxidans* have imaging findings similar to those of NTM infections. In the present case, although the patient was suspected to have NTM based on CT results, acid‐fast bacteria were not detected in the sputum or bronchial suction phlegm. In contrast, *A. xylosoxidans* persisted from the initial bronchoscopy. The number of cultured colonies also increased. As a result, a diagnosis of *A. xylosoxidans* infection was reached, and ceftazidime administration for 2 weeks improved the symptoms and imaging findings. Chronic infection with *A. xylosoxidans* should be considered in cases of suspected chronic airway infection in which NTM is not detected in multiple airway specimens.

In conclusion, *A. xylosoxidans* rarely cause chronic lower respiratory tract infections in patients with background lung disease. *A. xylosoxidans* may be a target for treatment when detected in the lower respiratory tract specimens after 2 weeks of ceftazidime administration.

## AUTHOR CONTRIBUTIONS

Dr. Kotani is the guarantor of this manuscript and contributed to the writing and review of the entire manuscript. Drs. Kasai, Suzuki, and Urushibara critically reviewed the manuscript. Drs. Inazaki and Rakuman collected and analysed the clinical data of the patients and critically reviewed the manuscript.

## CONFLICT OF INTEREST STATEMENT

None declared.

## ETHICS STATEMENT

Appropriate written informed consent was obtained from the patient for the publication of this case report and the accompanying images.

## Data Availability

Data sharing not applicable to this article as no datasets were generated or analysed during the current study.
